# Anatomical, histochemical, and immunohistochemical observations on the gastrointestinal tract of *Gallinula chloropus* (Aves: Rallidae)

**DOI:** 10.1186/s40850-022-00161-6

**Published:** 2022-12-28

**Authors:** Ahmed M. Abdellatif, Amany Farag, Elsayed Metwally

**Affiliations:** 1 Department of Anatomy and Embryology, Faculty of Veterinary Medicine, Mansoura University, Mansoura, 35516 Egypt; 2 Department of Cytology and Histology, Faculty of Veterinary Medicine, Mansoura University, Mansoura, 35516 Egypt; 3 Department of Cytology and Histology, Faculty of Veterinary Medicine, Suez Canal University, Ismailia, Egypt

**Keywords:** Apoptosis, Enteroendocrine cells, Gastrointestinal tract, Moorhen, Mucosubstances, Proliferation

## Abstract

**Background:**

Gallinula* chloropus* (Linnaeus, 1758) is a wild aquatic omnivorous bird characterized by a marked resistance to harsh environmental conditions and a worldwide distribution. In this study, anatomical, morphometrical, histochemical, and immunohistochemical techniques were employed to study the structure of the gastrointestinal tract of *Gallinula chloropus*.

**Results:**

The esophagus appeared tubular with no distinct crop. Both superficial (SPG) and deep (DPG) proventricular glands were present. The DPG filled about two-thirds of the total wall thickness. Histochemically, the mucosubstances revealed mixed alcian blue-PAS positive reactions. They were mainly localized in the acini of the esophageal glands and SPG, gastric surface epithelium, duct system of DPG, and intestinal goblet cells. The highest number of goblet cells per every 1 mm^2^ of the intestinal mucosa was seen within the ileum and rectum, 2555 ± 468 and 2607 ± 653 respectively. Notably, glucagon immunoreactive (IR) cells were abundant in the mucosa of the small and large intestines and the proventriculus, while somatostatin IR cells were concentrated within the acini of the DPG. IR cells for the mitosis marker phospho-histone H3 (PHH3) were highest within the entire intestinal crypts and mucosa-associated lymphoid tissues (MALT). In contrast, cells IR for the apoptosis marker C.CASP3 were remarkable in epithelial cells at the tips of intestinal villi and in MALT, reflecting the dynamic nature of the latter mentioned structures.

**Conclusions:**

The findings of the present study advance our knowledge of the gross and microscopic anatomy of the gastrointestinal tract in wild birds and could help to enhance the productivity of Aves via improving gut health.

## Background

Birds are among the most ecologically and evolutionarily conserved vertebrates as several avian species are able to survive across wide geographical regions of the world [1]. *Gallinula chloropus* (Linnaeus, 1758), also called common moorhen, Eurasian moorhen, or black gallinule, is a wild aquatic bird belonging to the family Rallidae was shown to withstand harsh environmental conditions [2]. It is the founding member of the genus Gallinula, one of the family's main genera [3]. The nests of *Gallinula chloropus* are frequently seen among reeds and vegetations covering the river banks and swamps [4]. These birds are sexually monomorphic with a characteristic red shield on their foreheads [5]. Common moorhens revealed a worldwide distribution especially in the Middle East, South and Central Asia, Europe, and North and South America [6]. Their migratory pattern occurs annually during winter from the north to south [7]. Fishermen hunt moorhens and their meat is well-known for its great taste. On the other hand, the transcontinental passage of these birds requires public health attention to limit the transmission of transborder diseases [8].

The gastrointestinal tract of most birds generally consists of four layers: the tunica mucosa, submucosa, muscularis, and adventitia or serosa [9]. A mucus layer of variable thickness covers the mucosa of different digestive tract parts that appear more remarkable within the large intestine. This layer is rich in mucosubstances or mucins that are known to play important roles in lubrication and protection of the underlying layers via limiting their exposure to toxins and pathogens [10]. Based on their pH, these mucosubstances are often classified as acidic or neutral [11]. The two main sources of mucins within the digestive tract are the glandular acini located within the deep face of the mucosa and goblet cells dispersed throughout the gut epithelium [12]. In most vertebrates the distribution and composition of digestive tract mucosubstances are species-specific. However, factors that alter the composition of the gut microbiota, e.g., diet or pathogenic bacteria, might affect them differently [13, 14].

Several neuroendocrine cells have been reported to be expressed by the mucosa of the vertebrate gastrointestinal tract [15]. Secretions of these cells are regulated by several factors including nutrients, enzymes, and hormones, and act mainly by exerting local effects on the neighboring cells [16]. Among the hormones secreted by the vertebrate digestive tract are glucagon and somatostatin. Unlike the well-organized cells of the endocrine organs, e.g., pancreatic islets in the pancreas, the enteroendocrine cells of the gastrointestinal tract are solitarily distributed among cells of the gut epithelium and revealed differential localization in the digestive tract of several studied avian species including chicken [17], ostrich [18], geese [19], and Passeriformes [20].

Programmed cell death, or apoptosis, and cell proliferation are two opposed, but intimately coupled, processes that occur simultaneously within several parts of the living body, though at different scales [21]. During apoptosis, the body selectively eliminates the undesired cells without provoking local damage [22]. Cell proliferation is a complex process that starts with a prolonged interphase and ends with brief mitosis [23]. Under physiological conditions, apoptosis and proliferation act together to ensure body homeostasis through balancing the tissues response to damaging insults they might encounter [24]. Altered apoptosis and proliferation rates are frequently seen in disease conditions affecting the avian digestive tract [25, 26]. Thus, mapping the normal distribution of apoptotic and/or proliferative cells within the avian digestive tract is a prerequisite for the proper diagnosis and treatment of such diseases [27].

Understanding the structure of the gastrointestinal tract will ultimately help to improve the digestion and productivity of birds. In the present study, different parts of the gastrointestinal tract of *Gallinula chloropus* from the esophagus to the rectum were collected and subjected to anatomical, histological, and histochemical analyses. In addition, the quantitative distribution of cells immunoreactive (IR) for glucagon and somatostatin as well as the mitosis marker phospho-histone H3 (PHH3), and the apoptosis marker cleaved caspase-3 (C.CASP3), was also studied using immunohistochemistry.

## Material and methods

### Birds

Sixteen (nine females & seven males) adult black gallinules (*G. chloropus*), weighing 210 ± 35 g, were safely captured by a professional hunter from stagnated regions in the vicinity of the Nile River at Dakahliya Governorate (Egypt). Six birds (three of each gender) were utilized for gross anatomy and morphometry. The rest of the birds were allocated to histology and immunohistochemistry. All birds were euthanized via cervical dislocation. Immediately after death confirmation, various digestive tract segments were exposed and fixed. All animal experiments complied with the ARRIVE guidelines and were performed according to the National Institutes of Health guide for the care and use of Laboratory animals (NIH Publications No. 8023, revised 1978). This study has been permitted by the Research Ethics Committee of the Faculty of Veterinary Medicine at Mansoura University, Egypt (VM.R.22.09.3.R1).

### Gross anatomy and morphometry

The viscera of each bird were reached via a ventral midline incision. All birds have been deemed healthy based on combined visual inspection of their external features and the appearance of their viscera. The length and width of all digestive segments were determined using a measuring tape as well as a digital vernier caliper (VINCA DCLA-0605, Clockwise Tools Inc., Valencia, CA, USA).

### Tissue sampling, histological, and histochemical analysis

The sampled parts of the moorhen digestive tract were the esophagus, the stomach (proventriculus, isthmus, and ventriculus or gizzard), the small intestine (duodenum, jejunum, and ileum), and the large intestine (ceca and rectum). Upon sampling, these parts were cleaned from debris using 0.9% saline solution, fixed in 10% neutral buffered formalin for 72 h, and embedded in paraffin. Four-μm-thick sections were cut and stained with the following stains: hematoxylin and eosin (H&E; for general histology), Masson's trichrome (for collagen and muscle fibers), Verhoeff–Van Gieson (for elastic, collagen and muscle fibers), periodic acid Schiff (PAS; for neutral mucins), alcian blue pH 2.5 (for acidic mucins), and combined alcian blue-PAS according to Woods, Stirling [28].

### Immunohistochemistry

Paraffin sections of 4 μm in thickness representing all parts of the digestive tract of the moorhen were prepared for DAB (3, 3'-diaminobenzidine) immunohistochemistry as previously described [29, 30]. Briefly, sections were dewaxed in xylene, washed in phosphate-buffered saline (PBS), and boiled in sodium citrate (pH = 6) inside a microwave for 20 min. Non-specific binding sites were then blocked via covering the sections with 5% bovine serum albumin/PBS for 1 h before applying the primary antibodies for another 3 h. Some sections incubated with the blocking buffer instead of the primary antibodies were set as negative controls. Following blocking of non-specific sites, biotinylated secondary antibodies (Jackson ImmunoResearch, West Grove, PA, USA) were applied to all sections for 30 min. The details of the antibodies used in the present study are listed in Table 1. The VECTASTAIN Elite ABC kit (PK-6100, Vector Laboratories, Burlingame, CA, USA) was used per manufacturer instructions to enhance the intensity of immunoreactions. The peroxidase activity of the sections was suppressed via incubating them with 0.3% H_2_O_2_/PBS for 30 min prior to application of the ABC kit. Immunoreactions were visualized using a freshly prepared DAB solution (SK-4103, Vector Laboratories). Finally, the nuclei of various cell types were counterstained with Mayer's hematoxylin, and the tissue sections were dehydrated and cleared.

**Table 1:** List of primary and biotinylated secondary antibodies used in the present study

Target protein	Primary antibody				Secondary antibody	
	**Clonality**	**Code #**	**Company**	**Dilution**	**Code #**	**Dilution**
GCG	Monoclonal	14–9743-80	Invitrogen (Waltham, MA, USA)	1:400	715–065-150	1:500
SST	Monoclonal	14–9751-80	Invitrogen (Waltham, MA, USA)	1:250	715–065-150	1:500
PHH3	Polyclonal	9701S	Cell signaling technology Inc. (Danvers, MA, USA)	1:200	711–065-152	1:500
C.CASP3	Polyclonal	9661S	Cell signaling technology Inc. (Danvers, MA, USA)	1:300	711–065-152	1:500

*C.CASP3* Cleaved caspase-3, *GCG* Glucagon, *PHH3* Phospho-histone H3, *SST* Somatostatin

### Photomicrography, image analysis, and cell counting

All stained sections were examined and photographed using an Olympus CH30 microscope (Olympus, Tokyo, Japan). Fifteen images per each studied part were randomly captured using the × 4, × 10, and × 40 objective lenses. All images were calibrated using a stage micrometer slide (MR095, Amscope, Irvine, CA, USA). The wall thickness of different segments of the moorhen digestive tract was determined in microscopic images taken using the × 4 objective lens. The average number of glucagon, somatostatin, PHH3, and C.CASP3 IR cells per the area of 1 mm^2^ of the mucosa of the esophagus, proventriculus, isthmus, ventriculus, duodenum, jejunum, ileum, ceca, and rectum were analyzed in microscopic images taken using the × 10 objective lens with the help of the area measurement and particle counting sections of the ImageJ software (National Institutes of Health, Bethesda, MD, USA) as previously described [31].

### Statistical analysis

Data were analyzed using GraphPad Prism 7 (GraphPad Software Inc.; San Diego, CA, USA). The distribution of glandular acini, goblet cells as well as the glucagon, somatostatin, PHH3, and C.CASP3 IR cells per each 1 mm^2^ of gastrointestinal segments from six birds (three per each gender) was compared using one-way ANOVA with Tukey's post hoc tests. *P*-values < 0.05 were used to express statistical significance.

## Results

### Gross anatomical findings

**Fig. 1:**
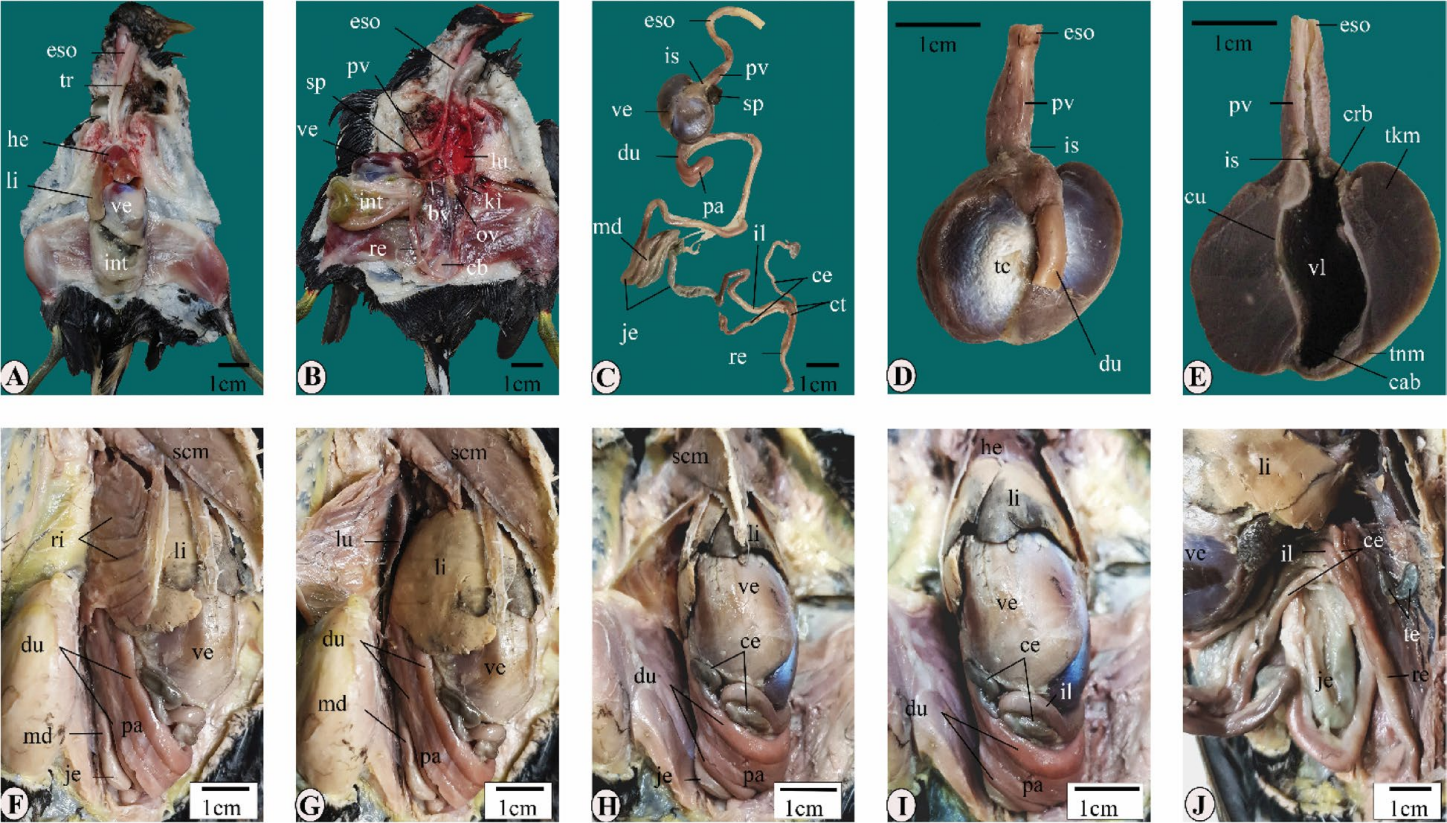
Gross anatomy of the gastrointestinal tract of the common moorhen. **A**, **B**) Dissected fresh specimens. **C**-**J**) Formalin-fixed specimens. **C**) Extracted whole gastrointestinal tract. **D**) Caudal esophagus, stomach, and cranial duodenum, intact. **E**) Caudal esophagus and stomach, sectioned. **F**-**J**) Appearance of the digestive organs on the right (**F**, **G**), ventral (**H**, **I**), and left (**J**) parts of the celomic cavity. Abbreviations: bv, intestinal blood vessels; cab, caudal blind sac; ce, ceca; cb, cloacal bursa; crb, cranial blind sac; ct, cecal tonsils; cu, cuticle; du, duodenum; eso, esophagus; he, heart; il, ileum; int, intestine; is, isthmus; je, jejunum; ki, kidney; li, liver; lu, lung; md, Meckel's diverticulum; ov, ovary; pa, pancreas; pv, proventriculus; re, rectum; ri, ribs; sp, spleen; scm; supracoracoideus muscle; tc, tendinous center; tkm, thick muscle; tnm, thin muscle; tr, trachea; ve, ventriculus; vl, ventricular lumen

The esophagus of the moorhen consisted of cervical and thoracic portions and was related to the right dorsal side of the trachea (Fig. 1A). No distinct crop was observed (Fig. 1B,C). The moorhen stomach was composed of the proventriculus and the gizzard (Fig. 1B-E). The moorhen stomach's two parts were separated by a short isthmus (Fig. 1D,E). The proventriculus was fusiform in shape and was related dorsally to the left lung, the left gonad, and the cranial pole of the left kidney. It was in contact ventrally and laterally with the left hepatic lobe and the spleen (Fig. 1A-D). The gastric isthmus was covered dorsally by the spleen (Fig. 1C). The ventriculus or gizzard appeared oval-shaped and represented the caudal and muscular portion of the moorhen stomach (Fig. 1A-I). Its wall revealed a well-developed tunica muscularis of variable thickness (Fig. 1E). A thick koilin layer lined the interior of the gizzard (Fig. 1E). The small intestine consisted of the duodenum, the jejunum, and the ileum. The duodenum appeared u-shaped with descending and ascending limbs enclosing the pancreas (Fig. 1C). The vitelline (Meckel`s) diverticulum was observed projecting from the jejunum at the right side of the celomic cavity (Fig. 1C,F,G). The large intestine was formed of the two ceca and the rectum (Fig. 1C). Prominent cecal tonsils appeared as mucosal thickenings close to the ileocecal junction (Fig. 1C). Each cecum appeared elongated with a pointed narrow tip (Fig. 1C). The two ceca filled part of the right ventral celomic cavity intervening between the caudal end of the gizzard and the duodenal loop (Fig. 1G-I). On the other hand, the rectum was located at the left caudal part of the celomic cavity (Fig. 1J).

### Morphometric, histological, and histochemical findings

**Fig. 2:**
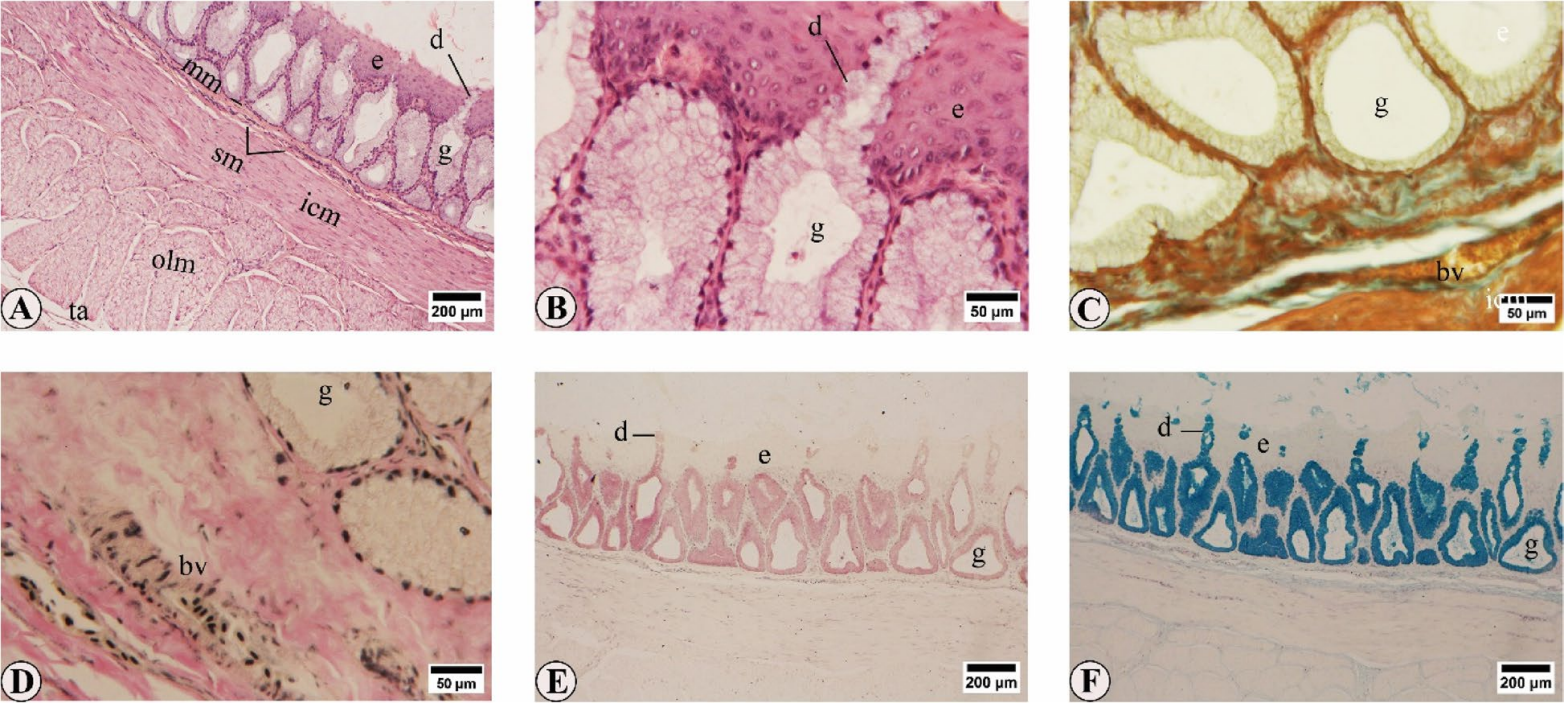
Histology and mucosubstances histochemistry of the wall of the esophagus in the common moorhen. **A**-**F**) Transverse sections through the esophageal wall stained with H&E (**A**, **B**), Masson`s trichrome (**C**), Verhoeff‐Van Gieson (**D**), PAS (**E**), and alcian blue-PAS (**F**). Abbreviations: bv, blood vessel; d, ducts; e, epithelium; g; esophageal glands; mm, lamina muscularis mucosae; sm, tunica submucosa; icm, inner circular layer of tunica muscularis; olm, outer longitudinal layer of tunica muscularis; ta, tunica adventitia

Morphometric measurements of the moorhen gastrointestinal segments are summarized in Table 2. The esophageal wall consists of four layers: the mucosa, the submucosa, the muscularis, and the adventitia or serosa (Fig. 2A). The lamina propria mucosae contained large amounts of mucus-secreting glands throughout the cervical and thoracic parts of the esophagus (Fig. 2A-F). These glands revealed an intense mixed positive reaction for both alcian blue and PAS and represented about one-fifth of the total wall thickness of the esophagus (Fig. 2E,F, Table 3). Their ducts perforated the overlying stratified squamous epithelium to reach the lumen (Fig. 2A,B). The tunica muscularis of the esophagus was formed of inner circular and outer longitudinal layers (Fig. 2A). The acini of the esophageal glands were almost equally reactive to both alcian blue and PAS staining (Fig. 2E,F).

**Table 2:** Morphometric measurements of the common moorhen gastrointestinal tract

Part		Length (mm)	Width (mm)	Wall thickness (µm)			
				**Mucosa**	**Submucosa**	**Muscularis**	**Adventitia/serosa**
Esophagus		90.3 ± 18.0	3.5 ± 0.6	609 ± 75	32 ± 7	1039 ± 198	122 ± 56
Stomach	PV	18.5 ± 3.7	6.4 ± 1.3	665 ± 84	1062 ± 175	495 ± 79	87 ± 21
	IS	3.1 ± 0.7	4.9 ± 0.9	714 ± 195		648 ± 146	92 ± 19
	VE	30.4 ± 5.4	20.3 ± 4.6	510 ± 93	75 ± 18	bs: 1107 ± 343 tnm: 8865 ± 960 tkm: 19,075 ± 1865	80 ± 17
Small intestine	DU	164.8 ± 36.2	2.9 ± 0.5	843 ± 112	19 ± 3.2	101 ± 13	18.2 ± 4.2
	JU	255.4 ± 58.7	3.3 ± 0.7	539 ± 56	18 ± 2.5	89 ± 16	19 ± 3.5
	IL	46.6 ± 7.0	3.2 ± 0.65	558 ± 71	23 ± 5.1	134 ± 14	21 ± 7.8
Large intestine	CE	69.4 ± 15.3	2.8 ± 0.55	458 ± 109	22 ± 4.3	301 ± 36	22 ± 3.9
	RE	41.9 ± 6.3	4.8 ± 5.2	493 ± 61	23 ± 4.3	370 ± 42	21 ± 5.1

*PV* Proventriculus, *IS* Isthmus, *VE* Ventriculus, *DU* Duodenum, *JU* Jejunum, *IL* Ileum, *CE* Cecum, *RE* Rectum, *bs* Blind sacs, *tnm* Thin muscles, *tkm* Thick muscles

**Table 3:** Distribution and nature of mucosubstances within the moorhen digestive tract

Part		GC (per 1 mm^2^)				GA
		**Total no. of GC**	**No. of mixed (alcian blue + PAS +) GC**	**No. of Acidic (alcian blue +) GC**	**No. of neutral (PAS +) GC**	**Area % from wall thickness**
Esophagus		-	-	-	-	21.4 ± 7.42^a^
Stomach	PV	-	-	-	-	65.6 ± 3.85^b^
	IS	-	-	-	-	30.3 ± 10.18^a^
	VE	-	-	-	-	24.6 ± 4.35^a^
Small intestine	DU	936 ± 172^a^	807 ± 164^a^	97 ± 21^a^	32 ± 11^a^	-
	JU	984 ± 119^a^	833 ± 122^a^	95 ± 26^a^	56 ± 9^a^	-
	IL	2555 ± 468^b^	2116 ± 438^b^	232 ± 54^b^	206 ± 50^c^	-
Large intestine	CE	2428 ± 161^b^	2218 ± 165^b^	129 ± 26^a^	81 ± 23^b^	**-**
	RE	2607 ± 653^b^	2407 ± 625^b^	110 ± 18^a^	90 ± 27^b^	-

Different superscript letters within the same column indicate statistical significance

*GC* Goblet cells, *GA* Glandular acini, *PAS* Periodic acid Schiff, *PV* Proventriculus, *IS* Isthmus, *VE* Ventriculus, *DU* Duodenum, *JU* Jejunum, *IL* ileum, *CE* Cecum, *RE* Rectum

**Fig. 3:**
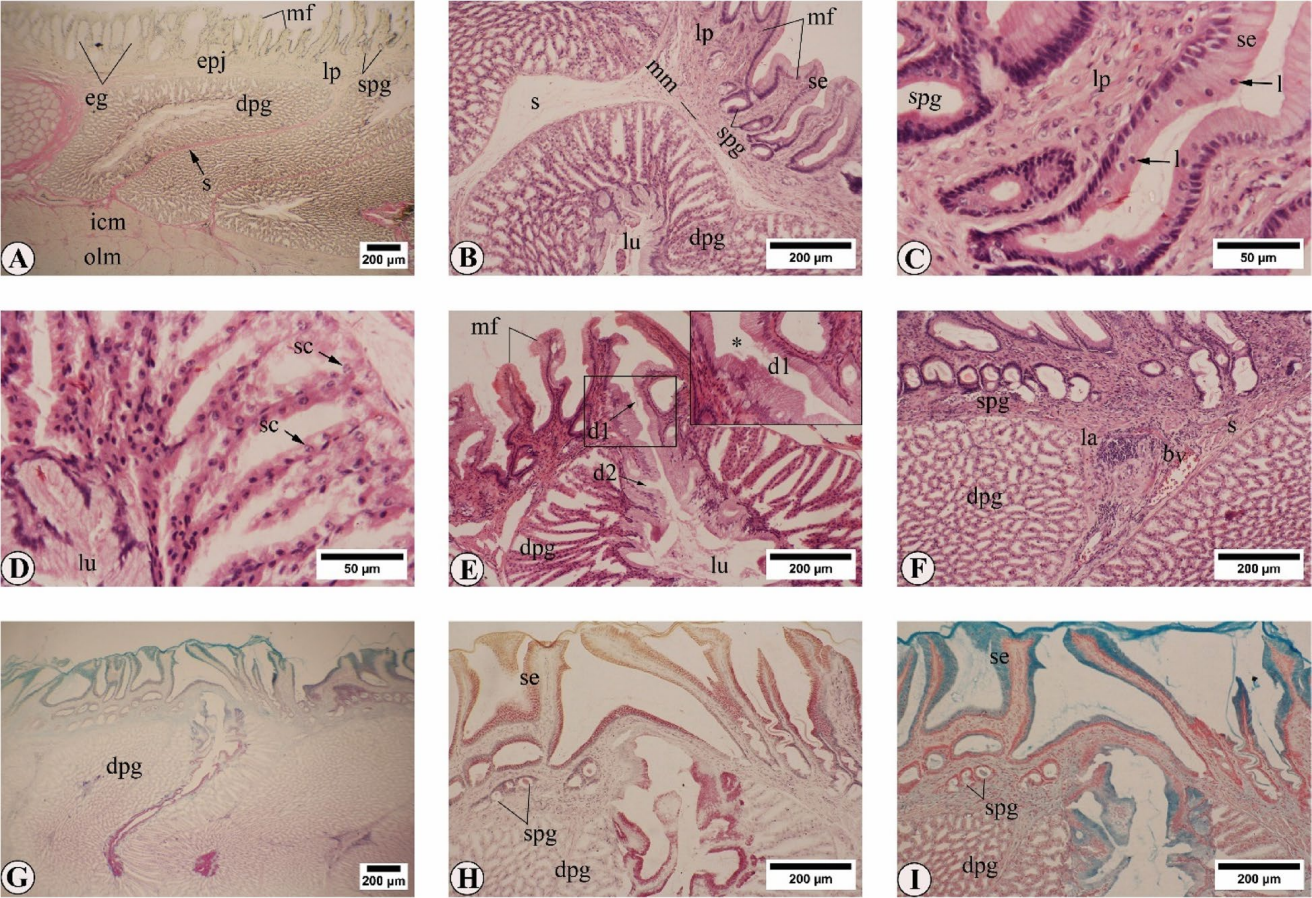
Histology and mucosubstances histochemistry of the wall of the proventriculus in the common moorhen. **A**-**F**) Sections through the proventricular wall stained with Verhoeff‐Van Gieson (**A**), H&E (**B**-**F**), alcian blue-PAS (**G**), PAS (**H**), and alcian blue (**I**). The area marked by an inset in Fig. 3E is magnified at the top right corner of the same figure. Abbreviations: bv, blood vessel; dpg, deep proventricular glands, the opening of one gland is denoted by an asterisk in Fig. 3E; d1, primary duct of dpg; d2, secondary duct of dpg; eg, esophageal glands; epj, esophago-proventricular junction; icm, inner circular layer of tunica muscularis, l, intraepithelial lymphocytes; lp, lamina propria; lu, lumen of dpg; la, lymphocytic aggregations; mf, mucosal folds, mm, lamina muscularis mucosae; olm, outer longitudinal layer of tunica muscularis; sc, secretory cells of dpg; s, septa; spg, superficial proventricular glands; se, surface epithelium

The mucosa of the proventriculus presented numerous folds (Fig. 3A-C). Each fold was formed of a core of connective tissue covered by a single cell layer of columnar epithelium (Fig. 3C). A number of lymphocytes was seen among the fold epithelium (Fig. 3C). Several lymphoid aggregations were also found within the lamina propria (Fig. 3F). The wall of the proventriculus showed two types of glands: the superficial glands with relatively small-sized acini, located within the mucosa and the deep glands with large-sized acini, situated within the submucosa (Fig. 3A-F). The deep proventricular glands occupied approximately two-thirds of the total wall thickness and separated from each other by connective tissue septae (Fig. 3A,F; Table 3).

The acini of the superficial proventricular glands as well as the surface epithelium of their overlying folds appeared predominately reactive for both alcian blue and PAS (Fig. 3G-I). On the other hand, the cells of the glandular units of the deep proventricular glands appeared neither reactive for alcian blue nor PAS (Fig. 3H,I). A prominent positive reaction for alcian blue and PAS staining was seen in the epithelial lining of the duct system of the latter mentioned glands (Fig. 3G, I). The openings of the ducts of the deep proventricular glands were observed between the mucosal folds, thus connecting the lumen of the gland to that of the proventriculus (Fig. 3D,E).

**Fig. 4:**
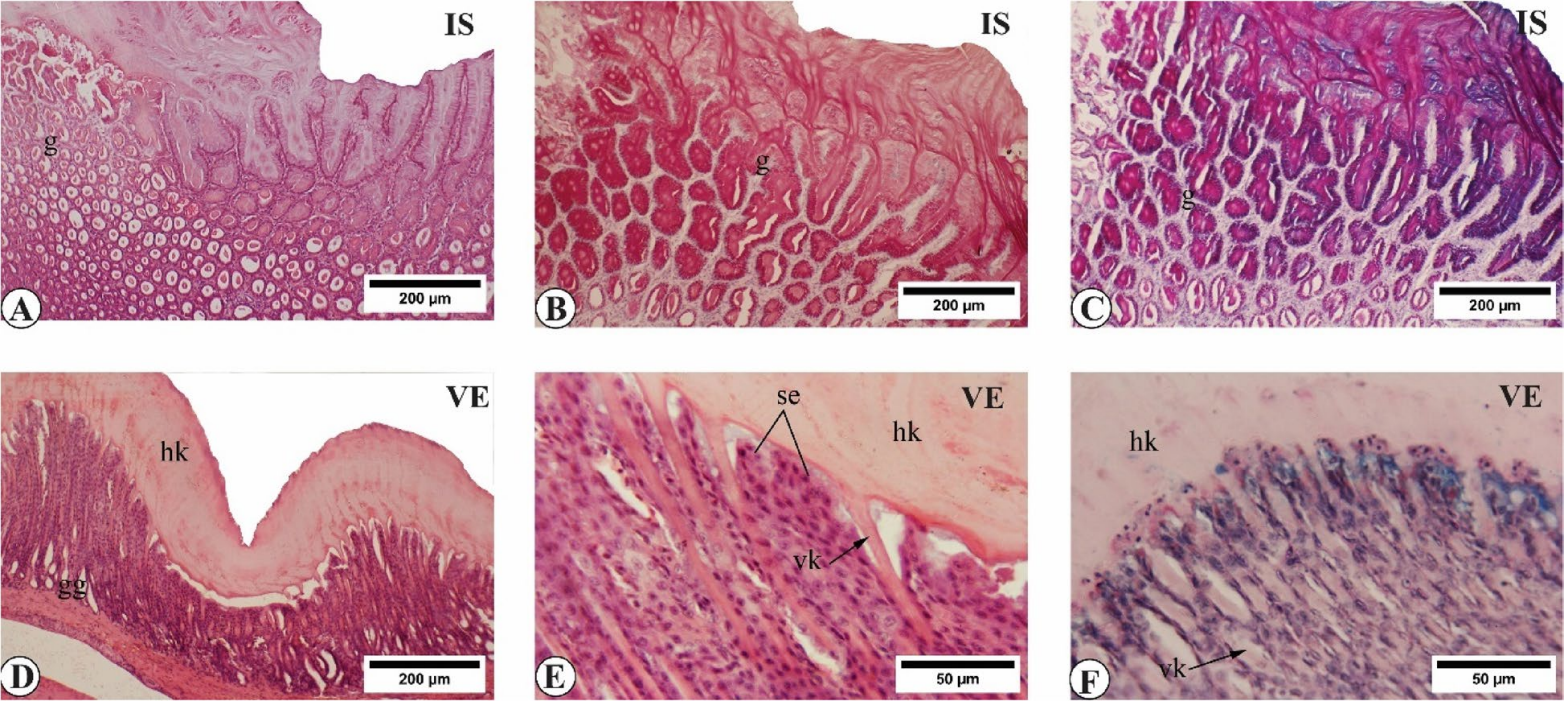
Histology and mucosubstances histochemistry of the wall of the isthmus and ventriculus in the common moorhen. **A-C**) Sections through the wall of the isthmus (IS) stained with H&E (**A**), PAS (**B**), and alcian blue-PAS (**C**). **D-F**) Sections through the wall of the ventriculus (VE) stained with H&E (**D,E**) and alcian blue-PAS (**F**). Abbreviations: gg, gastric glands; hk, horizontal koilin; g, isthmus glands; se, surface epithelium; tm, tunica muscularis; vk, vertical koilin

The short gastric isthmus was lined by a koilin layer that appeared poorly developed and lacked the submucosal glands. Instead, the mucosal glands of the isthmus appeared highly branched forming a peacock tail-like structure (Fig. 4A-C). The latter revealed a mixed alcian blue-PAS reaction with strong PAS affinity (Fig. 4B,C).

The gizzard was lined by a thick koilin layer which received the openings of the underlying tubular gastric glands (Fig. 4D-F). It was divisible into vertical parts filling the gastric pits and the glandular lumina and horizontal parts stretching on the surface of the gizzard epithelium (Fig. 4D-F). The surface epithelium of the gizzard revealed a mixed alcian blue-PAS reaction with strong alcian blue staining and weak to moderate PAS staining, whereas the acini of the gastric glands reacted weakly for both stains (Fig. 4F).

**Fig. 5:**
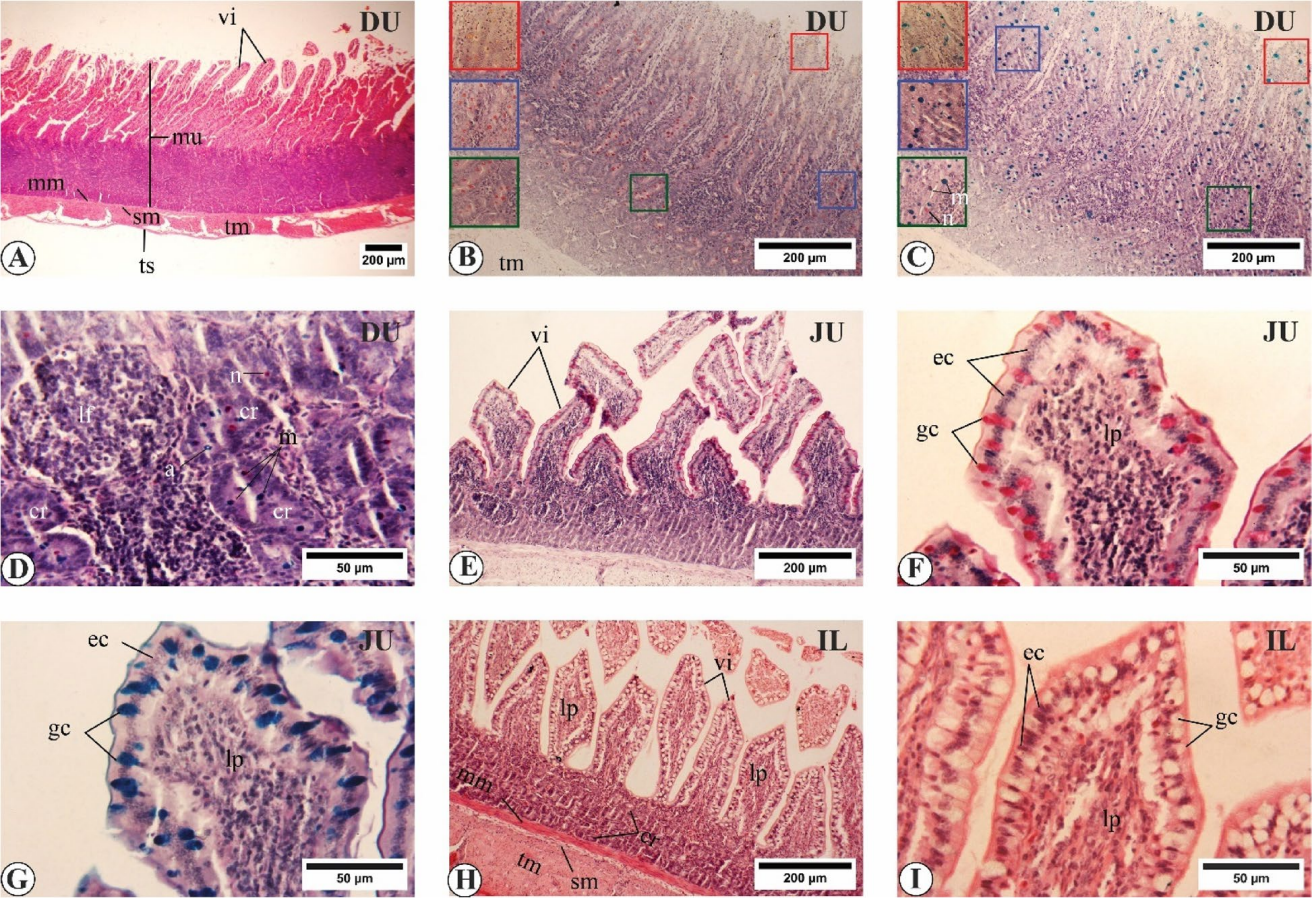
Histology and mucosubstances histochemistry of the wall of the small intestine in the common moorhen. **A**-**D**) Sections through the wall of the duodenum (DU) stained with H&E (**A**), PAS (**B**), and alcian blue-PAS (**C**, **D**). **E**–**G**) Sections through the wall of the jejunum (JU) stained with PAS (**E**, **F**), and alcian blue-PAS (**G**). **H**, **I**) Sections through the wall of the ileum (IL) stained with **H **&** E**. Abbreviations: a, acidic goblet cells; cr, crypts; ec, enterocytes; gc, goblet cells; lp, lamina propria; lf, lymphoid follicle; m, mixed goblet cells; mu, mucosa; mm, lamina muscularis mucosae; n, neutral goblet cells; sm, submucosa; tm, tunica muscularis; ts, tunica serosa; vi, villi

**Fig. 6:**
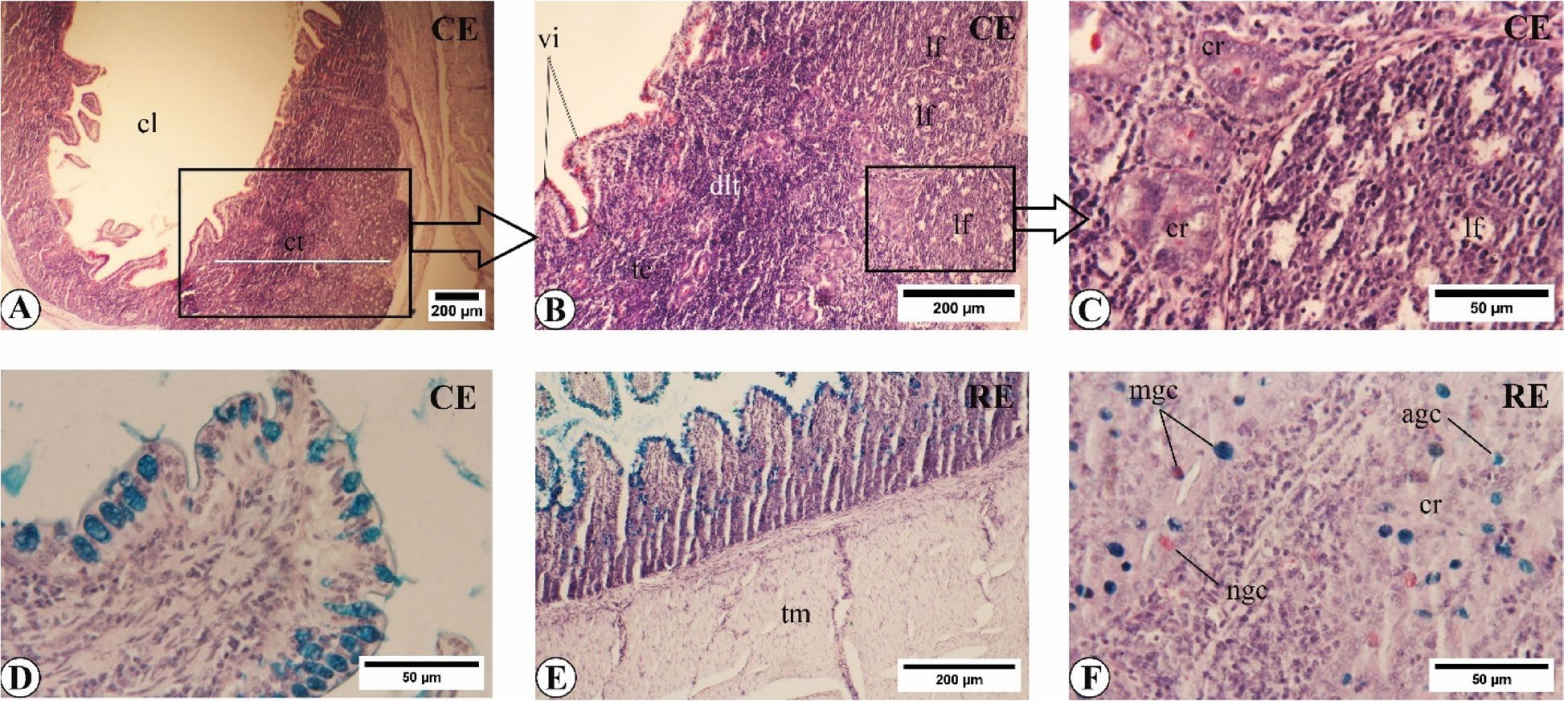
Histology and mucosubstances histochemistry of the wall of the large intestine in the common moorhen. **A**-**D**) Sections through the wall of the cecum (CE) stained with PAS (**A**-**C**), and alcian blue-PAS (**D**). **E**, **F**) Sections through the wall of the rectum (RE) stained with alcian blue-PAS. Abbreviations: agc, acidic goblet cells; cl, cecal lumen; ct, cecal tonsil; cr, crypts; dlt, diffuse lymphoid tissue; lf, lymphoid follicle; mgc, mixed goblet cells; ngc, neutral goblet cells; tc, tonsillar crypt; tm, tunica muscularis; vi, villi

Regarding the small intestine, the jejunum represented its longest segment followed by the duodenum, and ileum (Table 2). The wall of the small and large intestines consisted of four layers: the tunica mucosa, submucosa, muscularis, and serosa (Figs. 5,6). The mucosa showed well-developed villi and occupied the greatest portion of the small intestinal wall (Fig. 5A). Lymphoid follicles of 70–85 µm diameter were seen within the deep parts of the mucosa of both duodenum and jejunum (Fig. 5A-D). Each villus revealed a connective tissue core capped by a single-cell layer of columnar epithelium (Fig. 5E-I). The intestinal villi appeared of differing length and width between the three segments of the small intestine: they appeared long and narrow within the duodenum measuring about 610 × 70 µm, but shorter and wider within the jejunum, 325 × 106 µm, and ileum, 326 × 110 µm.

Concerning the large intestine, the cecum appeared longer but narrower than the rectum (Table 2). Compared to the small intestine, the mucosa of the large intestine presented slightly shorter and broader villi (Fig. 6A-F). The cecal tonsil was formed of a number of basally seated lymphoid follicles and an overlying diffuse lymphoid tissue that connects them to the surface epithelium (Fig. 6A-C). The thickness of the tunica muscularis of the large intestine was roughly three times that of the small intestine (Table 2).

The number of the intestinal goblet cells revealed a progressively increasing pattern from the duodenum toward the rectum (Figs. 5, 6, Table 3). The numbers of the goblet cells within the mucosa of the ileum appeared larger than these of the duodenum and jejunum. However, it was comparable to those of the cecum and rectum (Table 3). The majority of these goblet cells expressed both acidic and neutral mucins (Figs. 5G, 6D-F).

### Immunohistochemical findings

**Fig. 7:**
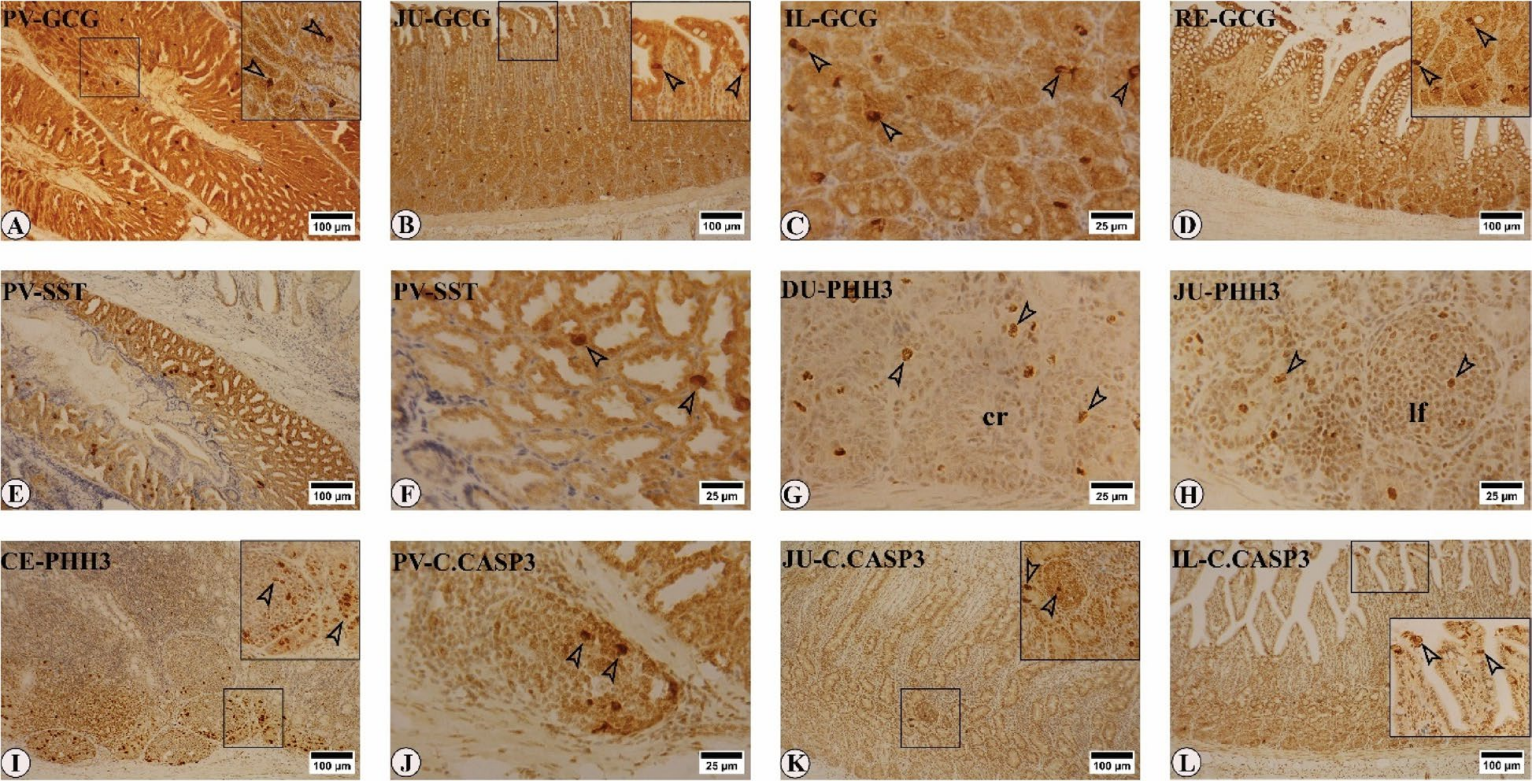
Representative figures for the distribution of glucagon (GCG, **A**-**D**), somatostatin (SST, **E**, **F**), phospho-histone H3 (PHH3, **G**-**I**), and cleaved caspase-3 (C.CASP3, **J**-**L**) immunoreactive (IR) cells throughout different parts of the common moorhen digestive tract. IR cells are indicated by empty arrowheads. PV, proventriculus; DU, duodenum; JU, jejunum; IL, ileum; CE, cecum; RE, rectum. Abbreviations: cr, crypts; lf, lymphoid follicles

Cells expressing glucagon, somatostatin, PHH3, and C.CASP3 were detected across the mucosa of different parts of the moorhen digestive tract with variable counts (Fig. 7, Table 4). The highest number of mucosal glucagon IR cells was detected in the crypts and villi of the small intestine followed by the acini of the deep proventricular glands (Fig. 7A-D). On the other hand, the somatostatin IR cells were concentrated mainly within the mucosa of the proventriculus especially the acini of the deep proventricular glands (Fig. 7E,F).

**Table 4:** Glucagon, somatostatin, phospho-histone H3, and cleaved caspase-3 immunoreactive cells per every 1 mm^2^ of different parts of common moorhen digestive tract

Part			GCG	SST	PHH3	C.CASP3
Esophagus			3 ± 1.3^a^	ND	32 ± 6.4^a^	28 ± 6.7^a^
Stomach	PV	SE	12 ± 3.5^b^	7 ± 1.65^b^	119 ± 13.7^d^	73 ± 15.5^b^
		SPG	10 ± 2.6^b^	13 ± 2.6^c^	112 ± 14.2^d^	71 ± 16.8^b^
		DPG	46 ± 10.1^c^	53 ± 11.0^d^	105 ± 7.6^d^	69 ± 18.3^b^
	IS		2 ± 0.8^a^	3 ± 0.9^a^	107 ± 14.3^d^	36 ± 10.6^a^
	VE		ND	ND	57 ± 10.5^b^	29 ± 8.5^a^
Small intestine	DU		76 ± 14.1^d^	ND	83 ± 4.9^c^	29 ± 6.0^a^
	JU		82 ± 16.9^d^	7 ± 1.9^b^	109 ± 12.8^d^	64 ± 13.7^b^
	IL		96 ± 17.3^d^	ND	84 ± 5.1^c^	35 ± 8.4^a^
Large intestine	CE		49 ± 10.8^c^	ND	128 ± 25.8^d^	83 ± 29.9^b^
	RE		47 ± 11.5^c^	ND	61 ± 7.2^b^	34 ± 8.9^a^

Different superscript letters within the same column indicate statistical significance

*C.CASP3* Cleaved caspase-3, *GCG* Glucagon, *PHH3* Phospho-histone H3, *SST* Somatostatin, *PV* Proventriculus, *SE* Surface epithelium, *SPG* Superficial proventricular glands, *DPG* Deep proventricular glands, *IS* Isthmus, *VE* Ventriculus, *DU* Duodenum, *JU* Jejunum, *IL* Ileum, *CE* Cecum, *RE* Rectum, *ND* Not detected

The proliferating PHH3 IR cells were confined mainly to crypts epithelium of the small and large intestinal segments as well as cells of the mucosa-associated lymphoid tissue (MALT) (Fig. 7G-I). The apoptotic cells as revealed by C.CASP3 immunostaining displayed a similar distribution pattern to that of PHH3 IR cells, though at a lower frequency (Table 4). A group of these cells was detected within goblet cells and enterocytes at the tips of the intestinal villi (Fig. 7J-L).

## Discussion

The structure of the gastrointestinal tract of the common moorhen was examined using anatomical, morpho-histometric, histochemical, and immunohistochemical methods. The anatomical study reports that the esophagus of the common moorhen consists of cervical and thoracic parts with no distinct crop. Differing from the studied species, the esophagus of several avian species including chicken, turkey, quail, pigeon, and dove forms a crop that is used by these birds to store and moisten food prior to its digestion [32]. A number of birds especially those of carnivorous feeding behavior including kestrel [33] and owl [34] have been reported to lack the crop. The crop`s functions in the latter Aves have been suggested to be taken over by the esophagus itself [35]. This probably applies also to the moorhen as both the mucus-secreting glands and the tunica muscularis of the esophagus appeared well-developed throughout its course. These structures might help in esophageal clearance and movement of food towards the stomach [36]. The wetting of the aquatic plants consumed by moorhen by the surrounding water might also help to replace the crop functions.

The wall of the moorhen proventriculus appeared in the present study containing superficial (mucosal) and deep (submucosal) proventricular glands. A similar presence of these glands has also been reported in the proventriculus of moorhen [37] and other avian species including the red jungle fowl [38], turkey [39] and hooded crow [40]. Additionally, the present study found that the deep proventricular glands filled a massive part of the proventricular wall, about two-thirds of the total wall thickness, suggesting a strong digestive ability for the studied species.

The histochemical staining of mucosubstances of the moorhen stomach revealed a mixed alcian blue-PAS positive reaction and they appeared mainly concentrated within the cells of the duct system of the deep proventricular glands. Similar reactions were also noted in the proventriculus of the grey-backed shrike [41]. Additionally and in line with our observations, the cells of deep proventricular glands appeared negatively reacting to alcian blue–PAS staining in the red jungle fowl [38] and the common starling [42]. The absence of significant alcian blue-PAS positive reactions in these cells suggests that the formation of mucosubstances in the moorhen glandular stomach is completed only near or at the ductal epithelium of the deep proventricular glands.

Goblet cells are the chief cells responsible for the production of intestinal mucus. Their number was found to increase in the intestine of moorhen on moving from the duodenum towards the rectum. A similar distribution pattern for goblet cells has also been recorded in common quail [43] and little owl [34]. The high number of goblet cells together with the relatively thicker tunica muscularis of the rectum probably act to facilitate the passage of undigested food to the exterior.

The present study revealed that most of the moorhen intestinal goblet cell population is of mixed type with a higher affinity to alcian blue than PAS staining. Consistent with these observations, a greater number of mixed goblet cells was observed in the intestine of broiler chicks [44, 45]. It is noteworthy that the timing of appearance of goblet cells in the aforementioned studies was found to be just before hatching with an earlier appearance of acidic mucus-producing goblet cells reflecting their importance in safeguarding the gut mucosa with the initiation of bird feeding.

Several hormones, including glucagon and somatostatin, are known to be expressed by the avian gastrointestinal tract. The hormone-expressing endocrine cells are dispersed among cells of the gastrointestinal epithelium and do not form aggregations like those found in the endocrine organs [46]. The glucagon and somatostatin IR cells of the moorhen digestive tract appeared of different shapes with intense cytoplasmic staining in the present study. The staining pattern of these cells agrees with previously published studies involving the digestive tract mucosa of several avian species [18–20]. The glucagon IR cells revealed the highest abundance within the small intestine followed by the proventriculus. A similar distribution of glucagon IR cells was reported in the digestive tract of goose [19]. However, such distribution of glucagon IR cells was different from that reported in rufous-collared sparrows where no glucagon IR cells were detected in the duodenum [20]. Literature about the role played by locally produced glucagon in avian gastrointestinal tract is scarce. Earlier reports in rat showed that infusion of enteric glucagon increased glucose absorption by intestinal cells more potently than that of pancreatic origin [47]. Recently, glucagon secretion by human gut has been proved to be evoked by high glucose concentrations (Sun et al., 2021), suggesting an evolutionarily conserved role for glucagon in the vertebrate gastrointestinal tract.

Regarding somatostatin expression, the present study showed the proventriculus as the major site of somatostatin IR cells within the moorhen digestive tract. Our findings were in line with those of Duritis, Mugurevics [18] in which a large number of somatostatin IR cells were observed within the proventriculus of ostrich. However, our findings were dissimilar from those reported by Mendes, do Nascimento [20] in rufous-collared sparrows where somatostatin IR cells were distributed at comparable numbers within the proventriculus, gizzard, and duodenum. The appearance of somatostatin IR in the aforementioned studies was mainly of closed type, i.e., having regular outlies with a limited number of cytoplasmic projections, that suggested a paracrine signaling effect of somatostatin without direct contact with their targets.

The epithelium of intestinal villi is characterized by its capacity for self-renewal under both normal homeostasis and damage-induced regeneration. In this regard, the intestinal crypts represent the main source for replenishing the damaged epithelial cells via continuous proliferation of their stem cell reserve [48]. Villus damage with decreased rates of crypt cell proliferation and increased rates of apoptosis is a hallmark of the malabsorption syndrome in chicks [25]. Using antibodies against phospho-histone H3 (PHH3) and cleaved caspase 3, the present study evaluated the rate of cell turnover within the moorhen gastrointestinal tract. These two markers are highly specific and widely used for identifying proliferating and apoptotic cells in clinical practice [49, 50]. We identified the crypt epithelium and the mucosa-associated lymphoid tissue (MALT) for cell proliferation and MALT and the tip of villus epithelium for apoptotic cells as the sites of moorhen intestinal mucosa with the highest cell cycle activity. The former structures included the proventricular lymphoid tissue, the lymphoid tissue in the wall of the duodenum and jejunum as well as the cecal tonsils. These sites covered most of the gastrointestinal tract and thus can be used as a basic reference for evaluating the gut immunity in the studied species and other birds and also for the diagnosis of diseases altering the gut cell cycle.

The present study suggests that the moorhen is an efficient cecal fermenter as its two ceca appeared long with gas distended tips. The two cecal tonsils present near the ileocecal junction are probably acting as immune guardians that ensure a balanced immune response within the caudal intestine. The high rate of turnover of immune cells within the lymphoid follicles of these tonsils revealed by PHH3 and C.CASP3 staining in the present study could reflect the dynamic status of these immune structures in the moorhen.

## Conclusion

The present study uncovered several aspects of the gross and microanatomic features of the moorhen gastrointestinal tract. Integrating data from the current work with those obtained from other avian species will help to increase our understanding of the factors governing digestion in wild birds and also to enhance the productivity of Aves via improving gut health.

## Data Availability

All data are available from the corresponding author upon request.
